# Differences in running biomechanics between young, healthy men and women carrying external loads

**DOI:** 10.3389/fbioe.2023.1250937

**Published:** 2023-10-03

**Authors:** Jose E. Rubio, Junfei Tong, Aravind Sundaramurthy, Adhitya V. Subramani, Vivek Bhaskar Kote, Michael Baggaley, W. Brent Edwards, Jaques Reifman

**Affiliations:** ^1^ Department of Defense Biotechnology High Performance Computing Software Applications Institute, Telemedicine and Advanced Technology Research Center, United States Army Medical Research and Development Command, Fort Detrick, MD, United States; ^2^ The Henry M. Jackson Foundation for the Advancement of Military Medicine, Inc., Bethesda, MD, United States; ^3^ Human Performance Laboratory, Faculty of Kinesiology, University of Calgary, Calgary, AB, Canada; ^4^ The McCaig Institute for Bone and Joint Health, Cumming School of Medicine, University of Calgary, Calgary, AB, Canada

**Keywords:** individualized models, load carriage, musculoskeletal injury, running, sex, stress fracture

## Abstract

During U.S. Army basic combat training (BCT), women are more prone to lower-extremity musculoskeletal injuries, including stress fracture (SF) of the tibia, with injury rates two to four times higher than those in men. There is evidence to suggest that the different injury rates are, in part, due to sex-specific differences in running biomechanics, including lower-extremity joint kinematics and kinetics, which are not fully understood, particularly when running with external load. To address this knowledge gap, we collected computed tomography images and motion-capture data from 41 young, healthy adults (20 women and 21 men) running on an instrumented treadmill at 3.0 m/s with loads of 0.0 kg, 11.3 kg, or 22.7 kg. Using individualized computational models, we quantified the running biomechanics and estimated tibial SF risk over 10 weeks of BCT, for each load condition. Across all load conditions, compared to men, women had a significantly smaller flexion angle at the trunk (16.9%–24.6%) but larger flexion angles at the ankle (14.0%–14.7%). Under load-carriage conditions, women had a larger flexion angle at the hip (17.7%–23.5%). In addition, women had a significantly smaller hip extension moment (11.8%–20.0%) and ankle plantarflexion moment (10.2%–14.3%), but larger joint reaction forces (JRFs) at the hip (16.1%–22.0%), knee (9.1%–14.2%), and ankle (8.2%–12.9%). Consequently, we found that women had a greater increase in tibial strain and SF risk than men as load increases, indicating higher susceptibility to injuries. When load carriage increased from 0.0 kg to 22.7 kg, SF risk increased by about 250% in women but only 133% in men. These results provide quantitative evidence to support the Army’s new training and testing doctrine, as it shifts to a more personalized approach that shall account for sex and individual differences.

## Introduction

Basic combat training (BCT) prepares new recruits to meet military-service requirements through a diverse set of strenuous physical activities, including running and foot marching. During U.S. Army BCT, overuse musculoskeletal injuries lead to lost-duty days and medical discharge, both of which affect military readiness (Hauret et al., 2001; Molloy et al., 2020). For example, Soldiers with a stress fracture (SF) require more than 60 days of recovery and rehabilitation before returning to duty, and more than 30% are discharged from the Army (Hauret et al., 2001). During BCT, the incidence of SF differs between men and women, with women having a fourfold greater injury risk than men (8% in women vs. 2% in men) (Knapik et al., 2012). Recently, Kardouni et al. (2021) reported a similar ratio of SF incidence between women and men during military training, with the highest rate observed between the third and eighth week of training. They also observed that the tibia is the most commonly affected injury site (35% of all SF in men and 19% in women), highlighting the importance of tibial SF as a medical impediment to Force readiness. As suggested by several studies (Ferber et al., 2003; Chumanov et al., 2008; Sinclair et al., 2012; Gehring et al., 2014; Sinclair and Selfe, 2015; Willson et al., 2015; Boyer et al., 2017), the examination of sex-specific differences in the kinematics and kinetics of running biomechanics offers the opportunity to gain insights into the etiology of lower-extremity musculoskeletal injuries and potentially create countermeasures to reduce the risk of these injuries.

Numerous studies have evaluated differences in joint biomechanics between men and women during running. For example, in terms of joint kinematics, women have greater hip adduction (Ferber et al., 2003; Willson et al., 2012; Gehring et al., 2014; Boyer et al., 2017), hip external rotation (Ferber et al., 2003), knee abduction (Ferber et al., 2003; Sinclair et al., 2012; Willson et al., 2012; Boyer et al., 2017), knee internal rotation (Sinclair et al., 2012), knee flexion (Boyer et al., 2017), and ankle eversion (Sinclair et al., 2012). In terms of joint kinetics, women have a larger external hip adduction moment (Gehring et al., 2014) but a smaller internal ankle plantarflexion moment (Boyer et al., 2017). However, some controversy exists concerning sex-specific differences in hip movement and knee extension moment during running. While Ferber et al. (2003) reported no difference in hip flexion angle between women and men, Boyer et al. (2017) found a greater hip flexion angle in young women (18–35 years old), and Sinclair et al. (2012) reported a smaller hip flexion angle in women. Similarly, for knee extension moment, Ferber et al. (2003) and Willson et al. (2015) found no differences between women and men, Boyer et al. (2017) determined it to be smaller in women, and Sinclair and Selfe (2015) reported it to be larger in women.

While different studies have separately investigated the running biomechanics of women or men when carrying load (Brown et al., 2014; Willy et al., 2016; Unnikrishnan et al., 2021; Tong et al., 2023), only a few have investigated sex-specific differences (Lobb et al., 2019; Brown et al., 2020; Wagers et al., 2022). Using individualized computational models that accounted for subject-specific anthropometric characteristics, tibial geometry and material properties, and gait patterns, our recent studies separately analyzed the joint mechanics of men and women when running with load, and suggest that there are sex-specific differences in hip flexion angle with increasing load (Unnikrishnan et al., 2021; Tong et al., 2023). In addition, Lobb et al. (2019) compared leg stiffness and ground reaction forces (GRFs) between women and men while running with loads ranging from 20 to 35 kg, and reported no sex-specific variations in response to these loads. In another study by the same group (Brown et al., 2020), they compared differences between women and men in lower-limb joint stiffness, range of motion, and peak flexion moment and found that women have a 15% stiffer knee than men when running with a load. Because an increase in knee joint stiffness facilitates the transmission of GRF to the joint itself and the lower-leg musculoskeletal system (Hamill et al., 2014; Brown et al., 2020), it is possible that the increased force on the proximal end of the tibia may cause greater tibial strains, leading to a higher risk of tibial SF (Unnikrishnan et al., 2021; Tong et al., 2023).

Although the aforementioned studies have contributed considerably to our understanding of how the biomechanics of men and women differ when running with and without load carriage, it remains unknown whether there are sex-specific differences in lower-extremity joint reaction forces (JRFs) and tibial strain during running with load carriage, as well as the extent to which these differences are associated with a risk of tibial SF. To address these knowledge gaps, in the current study we extended our previous work, which separately evaluated the running biomechanics of women (Unnikrishnan et al., 2021) and men (Tong et al., 2023), to directly assess sex differences under the same experimental conditions. Thus, the objective of this study is to quantify sex differences in the running biomechanics of young, healthy men and women as a function of load carriage. We hypothesize that women have higher joint reaction forces at the hip, knee and ankle, higher hip angles (flexion and extension), higher knee flexion angle, higher ankle angles (dorsiflexion and plantarflexion), larger hip external moments (flexion and extension), larger knee-extension external moment, larger ankle-plantarflexion external moment, higher trunk flexion angle, and higher tibial strain and SF risk than men, regardless of load condition. By delineating these dissimilarities, our study offers insights into biomechanical parameters that should be further evaluated for its association with SF risk. The knowledge gained in our study is key for the development of mitigation strategies against this injury, including personalized training programs that consider sex differences, and is in alignment with the new U.S. Army training doctrine described in the Holistic Health and Fitness publication (2020).

## Materials and methods

To achieve our objective, we collected experimental data while subjects ran with no load, an 11.3-kg load, and a 22.7-kg load, and then developed individualized musculoskeletal finite-element (FE) models to compute joint kinetics, tibial biomechanics, and SF risk for each subject under each load condition.

### Participants and data collection

We enrolled 42 young, healthy women (N = 21) and men (N = 21) representative of military recruits (18–21 years old), in accordance with an anthropometric survey of U.S. Army Soldiers (Gordon et al., 2014). All participants provided written informed consent and were self-reported experienced treadmill runners who exercised at least three times per week and free from injuries that could limit their physical activity for at least 3 months prior to enrollment in the study. For each participant, we recorded age and anthropometric measurements (Table 1) and collected computed tomography (CT) scans of the left leg, using a GE Discovery scanner (General Electric Medical System, Milwaukee, WI), with a slice thickness of 0.63 mm and an in-plane pixel resolution of 0.49 × 0.49 mm^2^.

**TABLE 1:** Anthropometric characteristics of young, healthy women and men in our study.

Group	Age (years)	Mass (kg)	Height (m)	Foot length (m)	Body fat (%)	BMI (kg/m^2^)
Women (N = 20)	19.7 (1.0)	60.3 (6.4)	1.65 (0.08)	0.24 (0.01)	18.3 (3.0)	22.2 (1.9)
Range	18–21	47.7–71.8	1.49–1.77	0.22–0.26	14.5–26.4	19.0–25.4
Men (N = 21)	19.6 (1.2)	72.0 (6.3)	1.77 (0.06)	0.27 (0.01)	8.7 (1.9)	23.0 (2.0)
Range	18–21	60.0–83.7	1.62–1.88	0.26–0.29	6.0–13.2	19.3–26.3
*t* statistic	−0.233	5.900	5.368	8.264	−12.054	1.285
*p*-value	0.817	**<0.001**	**<0.001**	**<0.001**	**<0.001**	0.207

The data are presented as means (one standard deviation) or range. A bold *p*-value indicates the parameter is significantly different between women and men, based on an unpaired *t*-test. BMI: body mass index.

Each participant completed running trials on an instrumented treadmill (Bertec Corporation, Columbus, OH) in a crossover, randomized order at a constant speed of 3.0 m/s under three load conditions [i.e., no load, an 11.3-kg load (25 lb), or a 22.7-kg load (50 lb)]. We selected the running speed and load carriages as representative of those during U.S. Army BCT (2020). We adjusted the load carriage using a V-max vest (V-max, Rexburg, ID), which distributed the load symmetrically between the front and back, because during BCT military personnel carry approximately symmetrical loads >90% of the time (Alemany et al., 2022).

At the start of the running trial, participants completed a 5-min warm-up period, consisting of walking at a self-selected speed on the treadmill. For each load condition, participants acclimated for an additional 30 s after achieving the prescribed speed. For each running trial, we synchronously collected motion-capture data at 200 Hz and force-platform data at 1,000 Hz, using an eight-camera motion-analysis system (Vicon Nexus, Centennial, CO) for at least 20 strides (∼20 s) after the participant reached a steady-state stride (i.e., a consistent stance and swing duration) at the prescribed speed. Specifically, we collected motion-capture data using 42 retroreflective markers secured bilaterally on the participant’s body, including anatomical landmark and segmental-tracking markers on the arm, trunk, pelvis, thigh, shank, and foot. Specifically, using Velcro and double-sided athletic tape, we placed markers on the anterior superior iliac crest, posterior superior iliac crest, greater trochanters, medial and lateral femoral condyles, medial and lateral malleoli, heel, and second and fifth metatarsals. In addition, we affixed segmental-tracking markers consisting of four-marker clusters on the anterior thigh and posterior shank. Between each trial, participants rested for as long as needed. While this short-duration experimental protocol did not capture the effects of musculoskeletal fatigue, it provided a sufficient number of strides for developing computational models.

The study protocol was approved by the University of Calgary Conjoint Health Research Ethics Board and by the Office of Human Research Oversight at the U.S. Army Medical Research and Development Command, Fort Detrick, MD.

### Computational analyses

The computational work included stride analysis, individualized musculoskeletal analysis, FE analysis, and SF risk prediction. We provided a detailed description of each of these computational elements in our previous works (Xu et al., 2016; Xu et al., 2020; Unnikrishnan et al., 2021; Tong et al., 2023). Briefly, for each participant under each load condition (running trial), we selected a representative stride using the GRF data and the method proposed by Sangeux and Polak (2015), which consists of the following six steps: *1*) identify the start and end points of each stride (and, hence, the stride duration) for the 20-s data by setting a threshold of 25 N for the vertical GRF (Tong et al., 2023); *2*) re-sample the GRF time history of each stride so that all strides of different durations are represented by 100 equidistant GRF values; *3*) determine the median GRF time history stride by calculating the median at each timepoint based on the re-sampled strides in the trial; *4*) for each re-sampled stride and timepoint, calculate the absolute difference between the GRF value and the corresponding median; *5*) for each re-sampled stride, compute the functional median distance (FDM) depth by integrating the calculated differences from *Step 4* over the stride duration; and *6*) select the stride with the smallest FDM value, which by default would be the stride closest to the median GRF time history stride, as the representative stride for a given trial. We then computed the stride duration and normalized stride length (i.e., normalized to body height) of the representative stride.

Using the motion-tracking data from the selected stride and the CT scans of the left tibia, we developed an *individualized* musculoskeletal model using the AnyBody Modeling System (AnyBody Technology, Aalborg, Denmark). Briefly, the software provides a generic musculoskeletal model of a male subject consisting of rigid segments, including arms, trunk, pelvis, thighs, shanks, and feet, as well as 55 muscles for each leg. Therefore, to develop an individualized musculoskeletal model, we first extracted subject-specific tibial geometry from the CT scans using an automated segmentation algorithm built in Mimics (Materialise, Leuven, Belgium) to segment the tibia, followed by a manual refinement to generate a clean (free of artifacts) tibial geometry, which we used to morph the tibial geometry in the generic human model available in AnyBody (Xu et al., 2016). Next, we scaled the other body segments of the generic model based on the subject’s height, body mass, foot length, and body fat percentage. Then, to further optimize the length and joint centers of the body extremities, we applied an optimization scheme to minimize the errors between the experimentally tracked and modeled marker positions (Andersen et al., 2010). Next, we applied a fourth-order Butterworth low-pass filter to the force-platform (20 Hz cutoff frequency) and marker-trajectory data (10 Hz cutoff frequency) (Xu et al., 2017; Baggaley et al., 2020). Finally, for each running trial of each subject, we used the musculoskeletal model to calculate the trunk flexion angle as well as the kinematics and kinetics of the lower-extremity joints exclusively for the representative stride. Specifically, in terms of kinematics, we computed the joint angles at the hip, knee, and ankle. In terms of kinetics, we calculated the resultant JRFs and external moments at the hip, knee, and ankle. We normalized the GRFs and JRFs by body weight and the joint moments by body mass.

To develop the *individualized* FE model, based on subject-specific tibial geometry extracted from the CT scans, we first generated a three-dimensional (3-D) mesh composed of 10-noded quadratic tetrahedral elements using the HyperMesh software (Altair Engineering, Inc., Troy, MI). Next, we used the Hounsfield units of the CT scans to determine the Young’s modulus (*E*) of each element, assuming that each element was linear elastic and isotropic. Based on the Young’s modulus, we classified each element as either intramedullary tissue (*E* < 6 MPa), trabecular bone (6 MPa ≤ *E* < 8 GPa), or cortical bone (*E* ≥ 8 GPa) (Rho et al., 1993). We assigned a Poisson’s ratio of 0.167 to the intramedullary tissue elements and 0.325 to the cortical and trabecular bone elements (Sandino et al., 2015). To conduct the FE analyses, we applied the muscle forces and joint forces/moments obtained from the musculoskeletal model as the loading conditions for the FE model, where we coupled the muscle and ligament insertion points to the outer surface of the tibial FE mesh. Using the Abaqus 2019 software (Dassault Systèmes, Vélizy-Villacoublay, France), we performed FE analysis and calculated the von Mises strain for each cortical bone element and determined the peak von Mises strain (90th percentile) of the entire cortical bone for the selected representative stride.

To predict the tibial SF risk for a simulated BCT regimen, we used a probabilistic model that accounted for bone fatigue damage, adaptation, and repair (Taylor et al., 2004), as detailed in our previous work (Tong et al., 2023). Briefly, the risk-prediction model used the tibial strain obtained from the FE model as input to determine the fatigue lifetime based on the S-N curve from a human tibia beam-bending experiment (Diab et al., 2005). To incorporate bone adaptation, we adjusted the tibial strain for each training day by multiplying it by a strain adaptation ratio using beam-theory equations, which assumed a bone deposition of 4 μm/day (Turner et al., 1994). Given the number of loading cycles per day, adjusted with a bone repair rate of 26 days, the model then predicted the tibial SF risk as a function of the number of training days (Taylor et al., 2004).

To determine the number of loading cycles per day, first, we mapped all strenuous activities (i.e., running and foot marching) during the 10-week BCT described in the Holistic Health and Fitness. (2020), which establishes the Army’s training and testing doctrine for achieving Soldier Readiness for 21st century warfare, into an equivalent running activity. Second, we estimated the total time of the equivalent running activity, defined a representative BCT week composed only of such running activity, and repeated it 10 times consecutively to simulate a 10-week BCT. The representative week consisted of five training days (1.7-km run per day) and two rest days. For each participant, we then divided the daily running distance by the participant’s representative load-condition-dependent stride length to calculate the number of loading cycles per day. Finally, we determined the tibial SF risk at the end of the 10th week of BCT for each participant under each loading condition.

### Statistical analysis

Prior to participant recruitment, we determined the sample size of each group (women and men) based on a power analysis. To calculate the sample size, we used means and standard deviations (SDs) of the peak angles and moments at the hip and knee of women and men while running with no load from a previous study (Ferber et al., 2003). Based on the calculated effect size (*ES*: 0.22), we determined that 21 participants for each group would be sufficient to show a statistical difference (*p* < 0.05) with a statistical power of 0.80.

We used an unpaired *t*-test to identify significant differences in age and anthropometric measurements between the two groups. To evaluate the difference in running biomechanics between women and men, for each dependent variable (e.g., stride duration, joint kinematics, and tibial SF risk), at each load condition, we performed a Mann-Whitney-Wilcoxon rank-sum test due to a small sample size (Fagerland, 2012) and calculated the *ES* (i.e., Cohen’s d) between women and men (Lakens, 2013). In addition, we applied the Benjamini–Hochberg correction in our analyses to account for multiple comparisons (Benjamini and Hochberg, 1995). Next, to assess whether load carriage affected women and men differently, we developed linear mixed-effects models for each dependent variable, where we included three fixed categorical effects (i.e., sex, load, and sex-load interaction) and a random intercept to account for within-subject variability. We then determined the significance of the interaction term using the Wald *F*-test with degrees of freedom adjusted based on the Kenward-Roger method (Luke, 2017). If the interaction effect was statistically significant, we calculated the *ES* between the no-load and 22.7-kg load conditions for women and men separately. Finally, we analyzed the tibial SF risk for the no-load and 22.7-kg load conditions using the empirical cumulative distribution function, which provided us the percentage of the predicted SF risk that was less than or equal to a specified value. We presented all data as means and SD, unless otherwise noted. We used the RStudio v1.4 statistical software (R Core Team, 2023) for all statistical analyses with an alpha level of 0.05.

## Results

We excluded one woman from our analysis because of loose markers during the motion-tracking experiment. Therefore, in total, our study included 20 women and 21 men. We found no significant differences between women and men in terms of age and body mass index (Table 1). However, compared to men, women had a significantly smaller mass, height, and foot length, and a significantly greater body fat percentage.

### Spatiotemporal parameters, trunk flexion angle, and joint kinematics

Except for the knee flexion angle, which we computed for the stance phase, all reported joint angles are peak angles for the entire stride. We found that women and men had a similar stance duration and normalized stride length regardless of load condition (Table 2). However, women had a significantly smaller peak trunk flexion angle than men under all three load conditions. The difference between women and men in the peak trunk flexion angle was 17.9% under the no-load condition [women: 16.8 (4.6) degrees vs. men: 20.1 (4.6) degrees, *ES*: 0.7], 24.6% under a 11.3-kg load [women: 17.5 (4.6) degrees vs. men: 22.4 (4.1) degrees, *ES*: 1.1], and 16.9% under a 22.7-kg load [women: 19.0 (3.9) degrees vs. men: 22.5 (3.9) degrees, *ES*: 0.9].

**TABLE 2:** Spatiotemporal parameters, peak trunk flexion angle, and peak joint angles at the hip, knee, and ankle in women and men while running with no load, an 11.3-kg load, and a 22.7-kg load.

Load	0.0 kg				11.3 kg				22.7 kg			
	Women (N = 20)	Men (N = 21)	*p*-value	*ES*	Women (N = 20)	Men (N = 21)	*p*-value	*ES*	Women (N = 20)	Men (N = 21)	*p*-value	*ES*
Stance duration (s)												
	0.25 (0.02)	0.26 (0.02)	0.337	0.5 (−0.2,1.1)	0.28 (0.02)	0.29 (0.02)	0.285	0.4 (−0.2,1.1)	0.29 (0.02)	0.3 (0.02)	0.289	0.4 (−0.2,1.0)
Normalized stride length												
	1.27 (0.09)	1.23 (0.07)	0.206	0.4 (−0.2,1.0)	1.25 (0.08)	1.23 (0.07)	0.488	0.3 (−0.3,0.9)	1.22 (0.07)	1.21 (0.07)	0.579	0.2 (−0.4,0.8)
Trunk flexion angle (degrees)												
	16.8 (4.6)	20.1 (4.6)	**0.027**	0.7 (0.1,1.4)	17.5 (4.6)	22.4 (4.1)	**0.004**	1.1 (0.5,1.8)	19.0 (3.9)	22.5 (3.9)	**0.016**	0.9 (0.2,1.5)
Hip angles (degrees)												
Flex	42.3 (7.7)	37.1 (7.3)	0.063	0.7 (0.1,1.3)	43.7 (7.1)	36.6 (6.0)	**0.004**	1.1 (0.4,1.7)	46.1 (6.4)	36.4 (6.6)	**<0.001**	1.5 (0.8,2.2)
Ext	19.4 (9.1)	21.8 (7.8)	0.367	0.3 (−0.3,0.9)	19.1 (9.9)	22.3 (7.7)	0.320	0.4 (−0.3,1.0)	18.6 (7.9)	22.4 (7.7)	0.206	0.5 (−0.1,1.1)
Knee flexion angle (degrees)												
	49.5 (5.1)	45.9 (5.2)	**0.028**	0.7 (0.1,1.3)	49.2 (5.0)	46.4 (5.0)	0.058	0.6 (−0.1,1.2)	49.2 (5.5)	46.0 (5.5)	0.074	0.6 (0.0,1.2)
Ankle angles (degrees)												
PF	18.0 (7.9)	17.6 (8.1)	0.558	0.1 (−0.6,0.7)	16.5 (8.6)	18.5 (7.7)	0.544	0.3 (−0.4,0.9)	15.4 (7.1)	17.7 (7.6)	0.434	0.3 (−0.3,0.9)
DF	42.1 (4.8)	36.6 (4.8)	**<0.001**	1.2 (0.5,1.8)	44.0 (4.9)	38.2 (5.3)	**<0.001**	1.1 (0.5,1.8)	45.2 (5.6)	39.0 (5.3)	**<0.001**	1.1 (0.5,1.8)

The data are presented as means (one standard deviation) or (95% confidence intervals). A bold *p*-value indicates a statistically significant difference between women and men, as determined by the Mann-Whitney-Wilcoxon rank-sum test. We applied the Benjamini–Hochberg correction in our analyses to account for multiple comparisons. DF, dorsiflexion; *ES*, effect size; Ext, extension; Flex, flexion; PF, plantarflexion.

**FIGURE 1:**
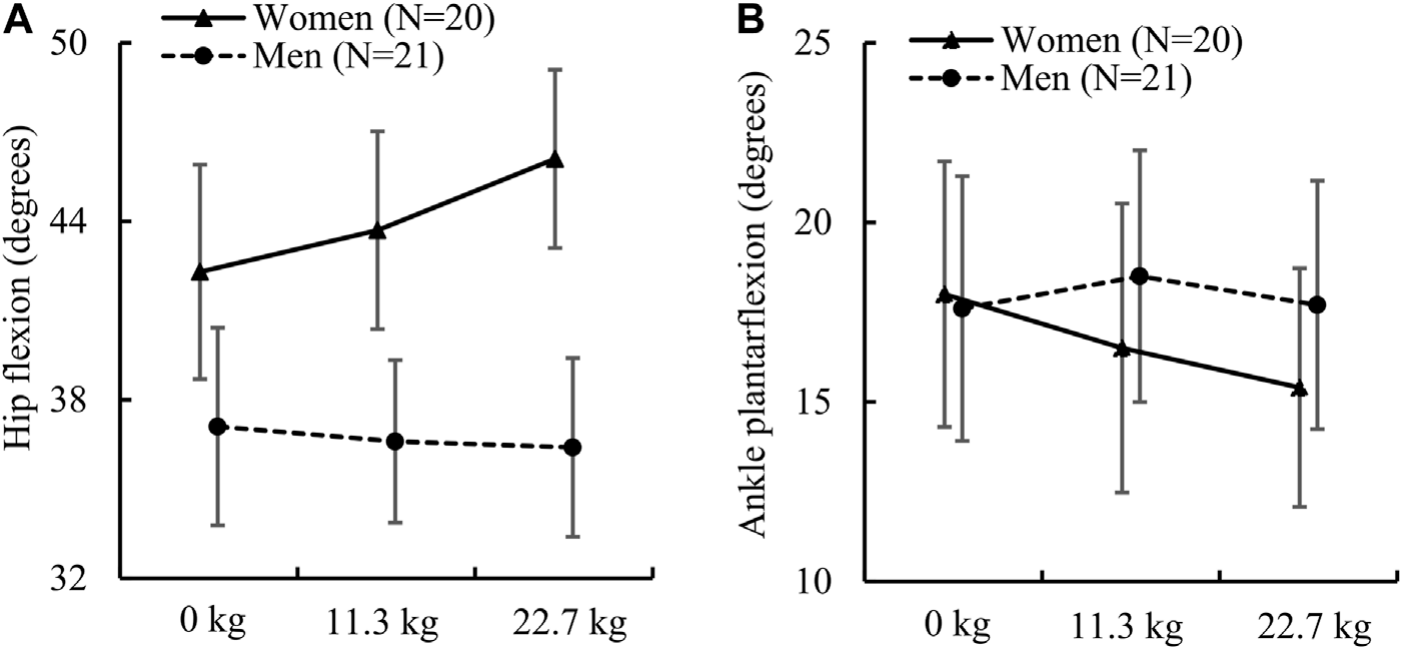
**(A)** Peak hip flexion angle and **(B)** peak ankle plantarflexion angle in women and men while running with no load, an 11.3-kg load, and a 22.7-kg load. The *p* values of the sex-load interaction effect for the peak hip flexion angle and ankle plantarflexion angle determined by the linear mixed-effects model were 0.002 [F (2) = 6.85] and 0.027 [F (2) = 3.80], respectively. Error bar: 95% confidence interval. F (df): F statistic (degrees of freedom).

For the hip joint kinematics, we found that women and men had a similar peak hip extension angle regardless of load condition. However, women had a significantly greater peak hip flexion angle than men under load-carriage conditions (Table 2). This difference was 17.7% under a 11.3-kg load [women: 43.7 (7.1) degrees vs. men: 36.6 (6.0) degrees, *ES*: 1.1] and 23.5% under a 22.7-kg load [women: 46.1 (6.4) degrees vs. men: 36.4 (6.6) degrees, *ES*: 1.5]. In addition, we also found that the sex-load interaction effect was statistically significant for the peak hip flexion angle (*p* = 0.002; Figure 1A). Specifically, compared to the no-load condition, a 22.7-kg load increased the peak hip flexion angle in women by 9.0% (*ES*: 1.0), but did not cause it to change in men. Herein, we only report the sex-load interaction effect *p*-value for the parameters that were statistically significant.

We found that the peak knee flexion angle in women was significantly greater than that in men only for the no-load condition. Nevertheless, the difference between women and men in the peak knee flexion angle varied from 5.9% to 7.5% for the three load conditions (Table 2). For the peak ankle plantarflexion angle, while women and men did not have significant differences regardless of load condition, we found that the sex-load interaction effect was statistically significant (*p* = 0.027; Figure 1B). Specifically, compared to the no-load condition, a 22.7-kg load decreased the peak plantarflexion angle in women by 14.4% [from 18.0 (7.9) degrees to 15.4 (7.1) degrees, *ES*: 0.6], but did not cause it to change in men. In contrast, we found that women had a significantly greater peak ankle dorsiflexion angle regardless of load condition, where the difference was 14.0% under the no-load condition [women: 42.1 (4.8) degrees vs. men: 36.6 (4.8) degrees, *ES*: 1.2], 14.1% under a 11.3-kg load [women: 44.0 (4.9) degrees vs. men: 38.2 (5.3) degrees, *ES*: 1.1], and 14.7% under a 22.7-kg load [women: 45.2 (5.6) degrees vs. men: 39.0 (5.3) degrees, *ES*: 1.1].

### Ground reaction force and joint kinetics

We found that women and men had similar peak normalized vertical GRFs, hip flexion moment, and knee extension moment regardless of the load condition (Table 3). However, women had a significantly lower peak hip extension moment and ankle plantarflexion moment than men under all three load conditions. For the peak hip extension moment, the difference between women and men was 20.0% under the no-load condition [women: 1.8 (0.2) N•m/kg vs. men: 2.2 (0.5) N•m/kg, *ES*: 1.2], 17.4% under a 11.3-kg load [women: 2.1 (0.3) N•m/kg vs. men: 2.5 (0.5) N•m/kg, *ES*: 1.0], and 11.8% under a 22.7-kg load [women: 2.4 (0.2) N•m/kg vs. men: 2.7 (0.4) N•m/kg, *ES*: 1.0]. For the peak ankle plantarflexion moment, the difference between women and men varied from 10.2% to 14.3%. In addition, by analyzing the temporal profiles of the GRFs, we estimated that 70% of women and 57% of men had a rear-foot-strike pattern.

**TABLE 3:** Peak ground reaction force, joint kinetics, tibial strain, and tibial stress-fracture risk in women and men while running with no load, an 11.3-kg load, and a 22.7-kg load.

Load	0.0 kg				11.3 kg				22.7 kg			
	Women (N = 20)	Men (N = 21)	*p*-value	*ES*	Women (N = 20)	Men (N = 21)	*p*-value	*ES*	Women (N = 20)	Men (N = 21)	*p*-value	*ES*
Ground reaction force (BW)												
	2.4 (0.1)	2.5 (0.2)	0.467	0.4 (−0.2,1.0)	2.6 (0.2)	2.6 (0.2)	0.325	0.4 (−0.2,1.0)	2.8 (0.2)	2.8 (0.3)	0.523	0.2 (−0.4,0.8)
Joint moments (N•m/kg)												
Hip Flex	0.9 (0.1)	0.9 (0.1)	0.615	0.0 (−0.6,0.7)	1.1 (0.2)	1.0 (0.1)	0.236	0.5 (−0.2,1.1)	1.2 (0.2)	1.1 (0.2)	0.203	0.4 (−0.2,1.0)
Hip Ext	1.8 (0.2)	2.2 (0.5)	**0.007**	1.2 (0.5,1.8)	2.1 (0.3)	2.5 (0.5)	**0.004**	1.0 (0.4,1.7)	2.4 (0.2)	2.7 (0.4)	**0.010**	1.0 (0.3,1.6)
Knee Ext	2.4 (0.5)	2.2 (0.4)	0.206	0.4 (−0.2,1.0)	2.4 (0.5)	2.3 (0.5)	0.388	0.2 (−0.4,0.8)	2.6 (0.5)	2.5 (0.5)	0.367	0.3 (−0.4,0.9)
Ankle PF	2.6 (0.3)	3.0 (0.5)	**0.007**	1.1 (0.4,1.7)	2.8 (0.2)	3.1 (0.5)	**0.005**	1.0 (0.4,1.7)	2.9 (0.3)	3.3 (0.5)	**0.010**	0.9 (0.3,1.5)
Joint reaction forces (BW)												
Hip	9.4 (1.5)	8.0 (1.5)	**0.007**	1.0 (0.3,1.6)	10.4 (1.7)	8.6 (1.4)	**0.004**	1.2 (0.5,1.8)	11.6 (1.4)	9.3 (1.5)	**<0.001**	1.5 (0.8,2.2)
Knee	12.6 (0.8)	11.5 (1.2)	**0.007**	1.0 (0.4,1.7)	13.8 (1.1)	12.5 (1.4)	**0.005**	1.0 (0.4,1.7)	15.1 (1.4)	13.1 (1.5)	**<0.001**	1.4 (0.7,2.0)
Ankle	12.7 (0.9)	11.7 (1.9)	0.140	0.6 (0.0,1.3)	13.7 (1.4)	12.4 (1.8)	**0.014**	0.8 (0.2,1.4)	14.9 (1.6)	13.1 (2.0)	**0.007**	1.0 (0.3,1.6)
Tibial strain (με)												
	5,007 (867)	4,540 (906)	0.188	0.5 (−0.1,1.1)	5,439 (1,042)	4,835 (1,045)	0.061	0.6 (0.0,1.2)	5,908 (1,159)	5,036 (1,191)	**0.031**	0.7 (0.1,1.4)
Tibial stress-fracture risk (%)												
	4.5 (5.5)	3.3 (6.0)	0.140	0.2 (−0.4,0.8)	8.9 (10.3)	5.6 (12.0)	**0.046**	0.3 (−0.3,0.9)	15.7 (16.9)	7.7 (14.4)	**0.031**	0.5 (−0.1,1.1)

The data are presented as means (one standard deviation) or (95% confidence intervals). A bold *p*-value indicates a statistically significant difference between women and men, as determined by the Mann-Whitney-Wilcoxon rank-sum test. We applied the Benjamini–Hochberg correction in our analyses to account for multiple comparisons. BW, body weight; *ES*, effect size; Ext, extension; Flex, flexion; PF, plantarflexion.

**FIGURE 2:**
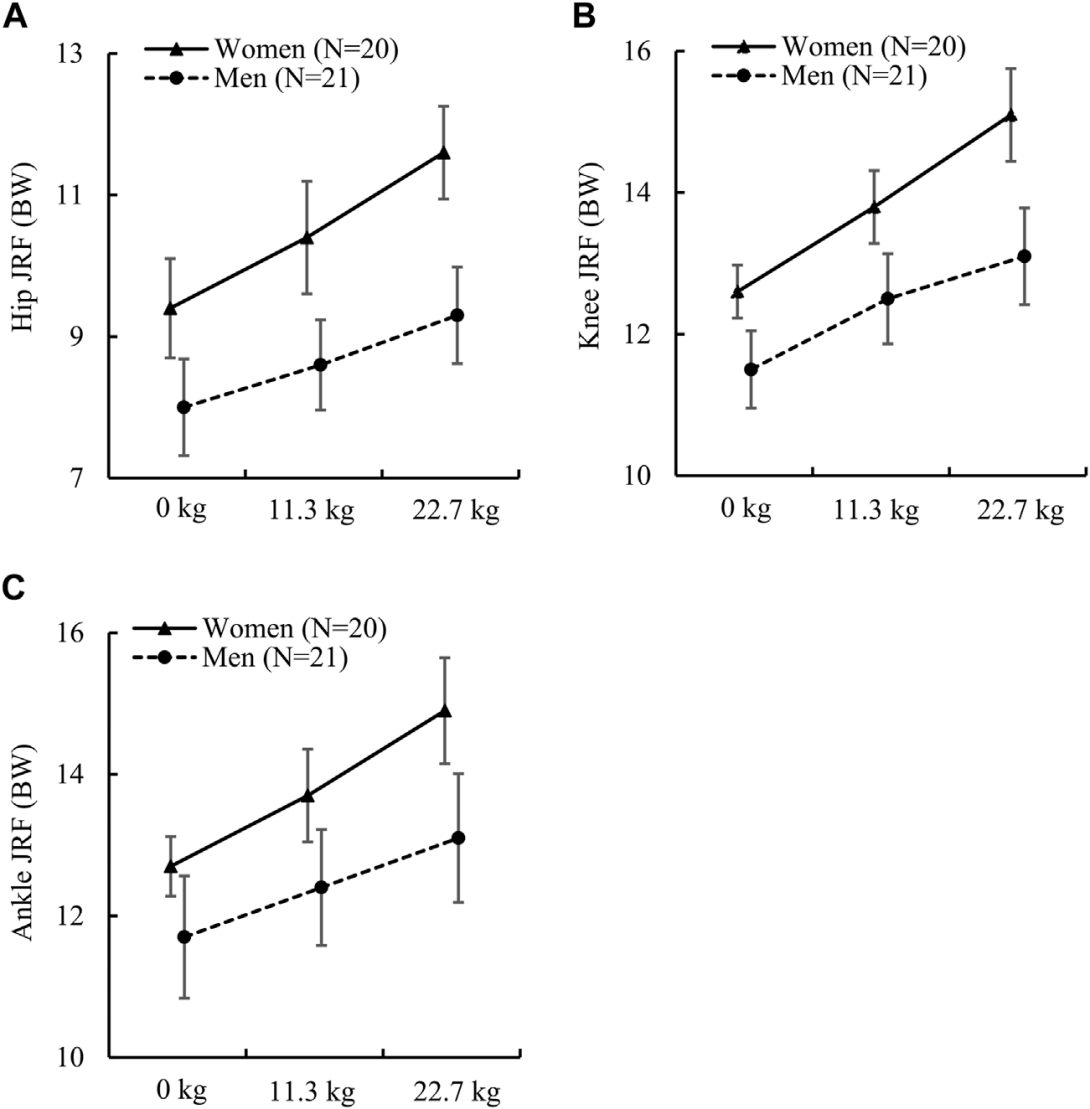
**(A)** Peak hip joint reaction force (JRF), **(B)** peak knee JRF, and **(C)** peak ankle JRF in women and men while running with no load, an 11.3-kg load, and a 22.7-kg load. BW: body weight. The *p* values of the sex-load interaction effect for the peak JRF at the hip, knee, and ankle determined by the linear mixed-effects model were 0.002 [F (2) = 6.73], 0.002 [F (2) = 6.90], and 0.015 [F (2) = 4.41], respectively. Error bar: 95% confidence interval. F (df): F statistic (degrees of freedom).

Unlike the joint moments, we found that women had significantly higher normalized JRFs at the hip, knee, and ankle under all load conditions except for the ankle JRF under the no-load condition, which had a *p*-value close to 0.05 and an *ES* of 0.6 (Table 3). For example, under the three load conditions, the difference between women and men varied from 16.1% to 22.0% for the hip JRF, from 9.1% to 14.2% for the knee JRF, and from 8.2% to 12.9% for the ankle JRF. In addition, we also found that the sex-load interaction effect was statistically significant for all three JRFs (Figures 2A–C). Compared to men, women had a larger relative increase in JRFs at the hip, knee, and ankle when the load increased. Specifically, compared to the no-load condition, a 22.7-kg load increased the hip JRF in women by 23.4% or 2.2 BW (*ES*: 2.5) but only 16.3% or 1.3 BW (*ES*: 1.9) in men. Similarly, a 22.7-kg load increased the knee JRF in women by 19.8% or 2.5 BW (*ES*: 2.5) but only 13.9% or 1.6 BW (*ES*: 1.9) in men, and increased the ankle JRF in women by 17.3% or 2.2 BW (*ES*: 2.4) but only 12.0% or 1.4 BW (*ES*: 1.6) in men.

### Tibial strain and stress-fracture risk

**FIGURE 3:**
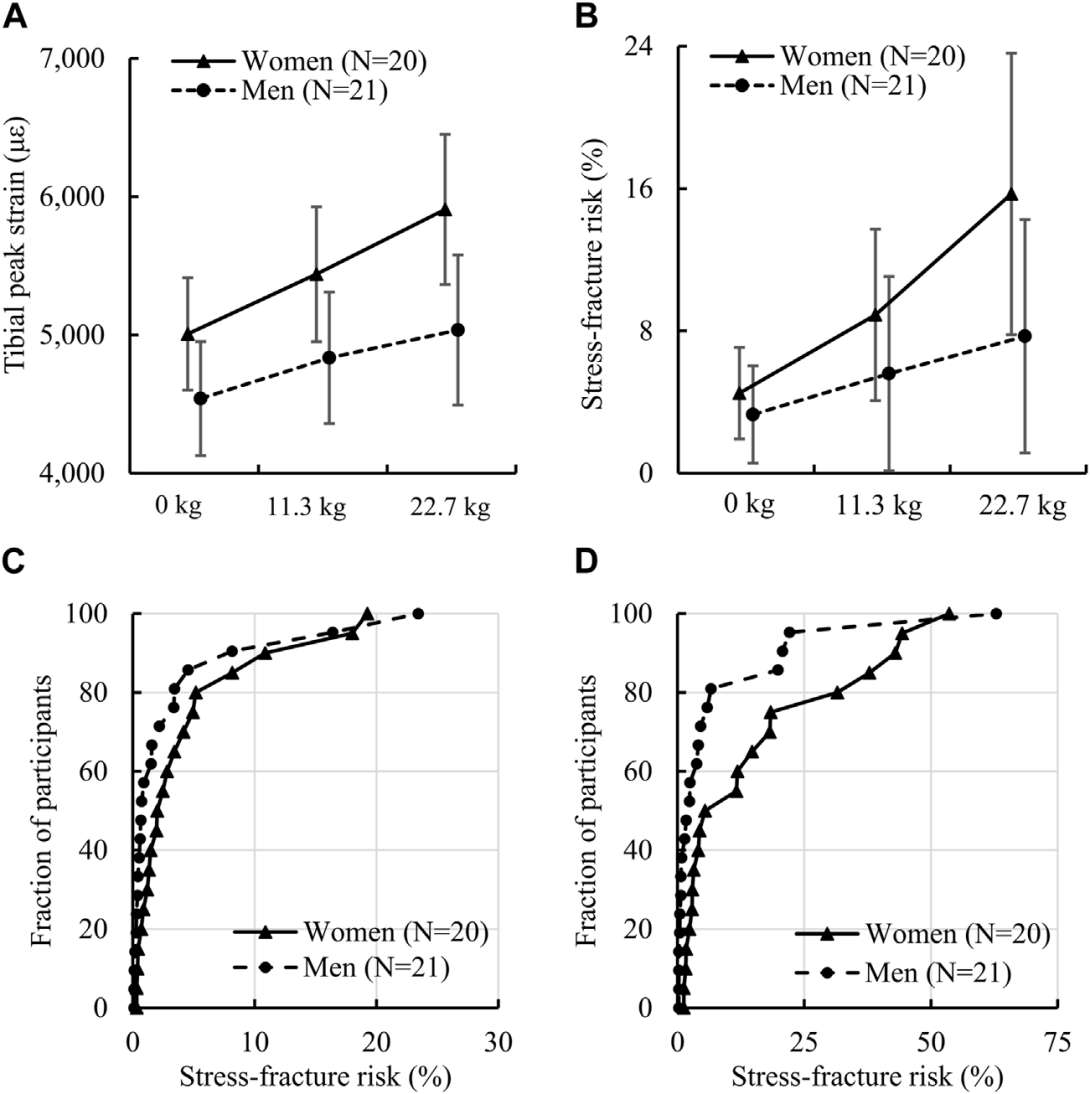
**(A)** Von Mises strain (90th percentile) of the tibia and **(B)** tibial stress-fracture risk in women and men while running with no load, an 11.3-kg load, and a 22.7-kg load. The *p* values of the sex-load interaction effect for tibial strain and stress-fracture risk determined by the linear mixed-effects model were 0.013 [F (2) = 4.55] and 0.046 [F (2) = 3.20], respectively. **(C)** Empirical cumulative distribution function of stress-fracture risk for the no-load condition and **(D)** empirical cumulative distribution function of stress-fracture risk for the 22.7-kg load condition. Error bar: 95% confidence interval. F (df): F statistic (degrees of freedom).

We found that women had a significantly higher peak tibial strain under the 22.7-kg load and higher SF risk under the 11.3-kg load or the 22.7-kg load but not under the no-load condition (Table 3; Figures 3A, B). Specifically, the difference between women and men in the peak tibial strain was 15.9% under a 22.7-kg load [women: 5,908 (1,159) με vs. men: 5,036 (1,191) με, *ES*: 0.7]. For tibial SF risk, the difference between women and men was 45.5% under a 11.3-kg load [women: 8.9 (10.3) % vs. men: 5.6 (12.0) %, *ES*: 0.3] and 68.4% under a 22.7-kg load [women: 15.7 (16.9) % vs. men: 7.7 (14.4) %, *ES*: 0.5]. In addition, we also found that the sex-load interaction effect was statistically significant for the peak tibial strain (*p* = 0.013; Figure 3A) and tibial SF risk (*p* = 0.046; Figure 3B). Specifically, compared to the no-load condition, a 22.7-kg load increased the peak tibial strain in women by 18.0% (*ES*: 1.7), but only 10.9% (*ES*: 1.0) in men, and the tibial SF risk by 249% (*ES*: 0.9), but only 133% (*ES*: 0.5) in men (Table 3). In addition, the empirical cumulative distribution function for SF risk showed that, for the no-load condition, the percentage of participants with a SF risk ≤5% was 75% for women, but 86% for men (Figure 3C). Similarly, for the 22.7-kg load condition, the percentage of participants with a SF risk ≤5% was 45% for women, but 71% for men (Figure 3D).

## Discussion

In the present work, our goal was to investigate differences in running biomechanics between young, healthy women and men under different load-carriage conditions. Towards this goal, we collected experimental data and developed computational models to assess the joint kinematics and kinetics, tibial biomechanics, and SF risk of 20 women and 21 men. We hypothesized that women would have larger joint (hip, knee, and ankle) angles, forces, and moments, increased trunk flexion angle, and higher tibial strain and SF risk than men, regardless of load condition. Although women and men had significantly different anthropometric characteristics in terms of height and mass (Table 1), in partial agreement with our hypothesis, we found that they had similarities in some running biomechanical parameters regardless of load condition, while for others they had significant statistical differences (Tables 2, 3; Figures 1–3).

Regardless of load condition, we found that women and men had equivalent stance duration, normalized stride length, peak hip extension angle, peak GRF, peak hip flexion moment, and peak knee extension moment, partially rejecting our hypothesis that women have larger joint angles, forces, and moments than men (Tables 2, 3). Prior studies that investigated the impact of sex on running biomechanics under a no-load condition corroborate our findings (Keller et al., 1996; Ferber et al., 2003; Schache et al., 2003; Sinclair et al., 2012; Boyer et al., 2017). For example, these studies found no significant differences between women and men in stance duration (Ferber et al., 2003; Sinclair et al., 2012), normalized stride length (Schache et al., 2003), hip extension angle (Boyer et al., 2017), peak GRF (Keller et al., 1996), hip flexion moment (Boyer et al., 2017), and knee extension moment (Ferber et al., 2003; Willson et al., 2015). However, it should also be noted that other studies reported opposite findings regarding the knee extension moment under the no-load condition. For instance, Boyer et al. (2017) found it to be smaller in women, whereas Sinclair and Selfe (2015) found it to be larger in women. We speculate that such discrepancies may be due to the uncertainties of the body-segment parameters (Riemer et al., 2008). In our study, we developed individualized musculoskeletal models through an optimization scheme that incorporated subject-specific anthropometric data and bone geometry in an attempt to overcome this limitation. Moreover, while most studies only investigated the no-load condition, we also included a load-carriage condition and found that the impact of load on these parameters was equivalent in women and men. The similarities between men and women in terms of stance duration, normalized stride length, peak hip extension angle, peak GRF, peak hip flexion moment, and peak knee extension moment (Tables 2, 3) suggest that these parameters are unlikely to be associated with the different incidence of tibial SF between sexes (Hollander et al., 2021).

We found that, regardless of load condition, women had a significantly smaller trunk flexion angle than men, partially rejecting our hypothesis that women have larger joint angles than men, but significantly larger hip flexion angle in agreement with our hypothesis, and a marginally larger knee flexion angle (Table 2). Prior studies have shown that both a large trunk flexion angle as well as large hip and knee flexion angles reduce the load on the knee extensor muscles but increase the load on the hip extensor muscles (Pollard et al., 2010; Teng and Powers, 2014; Warrener et al., 2021). For example, Teng and Powers (2014) and Warrener et al. (2021) found that an increase in the trunk flexion angle during running significantly reduces the knee extensor moment but increases the hip extensor moment for both women and men. Meanwhile, Pollard et al. (2010) found that women who have larger hip and knee flexion angles during a drop-landing task experience a significantly lower knee extensor moment and a greater hip extensor moment. In addition, the larger hip and knee flexion angles in women and the larger trunk flexion angle in men indicate that women and men adopt different strategies to partially shift the load from the knee extensor muscles to the hip extensor muscles. We speculate that this may be due to the anatomical differences in the lumbar vertebrae between women and men. Specifically, women have dorsal wedging in three vertebrae (i.e., L3, L4, and L5), whereas men only have it in the L4 and L5 vertebrae (Whitcome et al., 2007). The additional vertebra with dorsal wedging enables women to increase their lumbar lordosis with less intervertebral rotation, which significantly reduces the shear force across the lumbar vertebral joints. However, this may also lead to a greater compressive force at the vertebrae (L5-S1) in women than in men under an equivalent trunk flexion (Bae and Mun, 2010; Bailey et al., 2016). In addition, women have a 25% smaller vertebral cross-sectional area than men, which is expected to cause a 30%–40% higher vertebral stress under an equivalent load (Gilsanz et al., 1994). Therefore, we speculate that, compared to men, women tend to flex their hip and knee more than their trunk during running to avoid overloading the vertebrae.

Our analysis of sex-load interaction revealed that load carriage affected men and women differently in terms of hip flexion angle (Figure 1A). Although we did not conduct a statistical analysis to evaluate within-sex differences due to external load, we found that women, but not men, increased their hip flexion angle by up to 9% with load carriage (Figure 1A). We speculate that the cause of this discrepancy is the difference in hip extensor muscle strength between men and women. When compared to men, women have a much weaker hip extensor muscle (Stearns et al., 2013) and increased hip extensor muscle activity when running with no load (Willson et al., 2012). Therefore, based on our results (Table 2), we speculate that women increased both their trunk flexion and hip flexion angles to maximize the output from the hip extensor muscle when carrying load. In contrast, men only needed to increase their trunk flexion angle as they have a stronger hip extensor muscle.

We found that, regardless of load condition, women and men had an equivalent plantarflexion angle, partially rejecting our hypothesis (Table 2; Figure 1B). However, when the load increased from 0.0 kg to 22.7 kg, women, but not men, reduced the ankle plantarflexion angle by 14.4%. A recent study by Moore (2016) suggests that this less-extended running style in women, with less plantarflexion and greater knee flexion, has a better running economy. From another perspective, this may also indicate that women experienced a greater burden when carrying an extra 22.7-kg load, as they had to make greater adjustment in their gait to accommodate the load. In addition, we found that women had a significantly larger dorsiflexion angle (Table 2). Prior studies reported similar findings, with women exhibiting larger dorsiflexion angles during running (Bazuelo-Ruiz et al., 2018) and drop landing (Kernozek et al., 2005). One possible explanation for such a sex-specific difference could be due to differences in ankle stiffness (defined as torque increment per unit ankle rotation) between women and men at different ankle positions. For example, Riemann et al. (2001) found that women and men had a similar ankle stiffness under a 10-degree plantarflexion, whereas under a 10-degree dorsiflexion, stiffness was more than 30% greater in men. When running with no load, men had a 15% larger ankle plantarflexion moment than women (Table 3), moreover, their disproportionately greater ankle stiffness still led to a smaller dorsiflexion angle (Table 2).

In support of our hypothesis, for normalized JRFs at the hip, knee, and ankle, we found that all JRFs were significantly larger in women regardless of load condition (Table 3; Figure 2), except for the ankle JRF at the no-load condition. These findings suggest that, under the same load carriage, women had greater localized loadings at the lower-extremity joints. In addition, we observed a significant sex-load interaction for the JRFs, indicating that load carriage affected men and women differently in terms of contact forces exerted on the hip, knee, and ankle joints. This may be due to the shorter muscle moment arms associated with the shorter body size (e.g., height and foot length) in women compared with men (Sheehan, 2012), which caused a larger increase in muscle forces and JRFs in women as the load increased.

In support of our hypothesis, our predicted SF risk after a 10-week BCT were larger in women (Table 3; Figures 3A, B). For all load conditions, we predicted that the SF risk after a 10-week BCT ranged from 4.5% to 15.7% in women and from 3.3% to 7.7% in men. This agrees well with a prior study by Knapik et al. (2012), who estimated the SF risk for women to range between 1.1% and 18.0% and for men between 0.8% and 5.1% during BCT. In addition, our SF-risk estimates (Figures 3C, D) align with clinical observations that the incidence rate of tibial SF in women is considerably higher than that in men during BCT (Kucera et al., 2016; Kardouni et al., 2021). The reasonable agreement between our predictions and the clinical observations suggests that individualized computational models, which incorporate subject-specific anthropometric characteristics, tibial geometry and material properties, and gait patterns, can adequately predict tibial biomechanics and SF risk.

Taken together, our results provide further evidence that variations in running biomechanics between men and women may lead to markedly different tibial SF risk. In addition, although our analyses do not account for the effect of musculoskeletal fatigue, our findings support the notion that training programs during BCT should account for sex differences to minimize the risk of injury. For example, while load carriage during BCT or military operations is inevitable, personalized training programs could be adapted to consider lighter load carriage, slower running speed (Meardon et al., 2021), and adoption of a shorter stride length (Edwards et al., 2009). This sex-specific training approach is in accordance with the U.S. Army Holistic Health and Fitness doctrine (2020), which aims to optimize Warfighter performance and reduce injury rates by following an individualized evidence-based training regimen.

Our study has several limitations. First, for both women and men, we performed all running trials at 3.0 m/s, while a prior study suggested that running speed impacts tibial biomechanics differently in men and women (Meardon et al., 2021). Future work on women running with different loads at different speeds would provide additional insights to optimize training programs for women. Second, we only assessed the acute impact of load on the running biomechanics while disregarding the effect of muscle fatigue, as women and men may fatigue differently while performing the same physical activities (Hunter, 2016). Therefore, future studies involving prolonged running should provide additional information on the impact of fatigue. Third, the study participants may not have had a history of load carriage, which could have influenced their biomechanics when running with load. Despite this uncertainty, we still consider the participants to be a representative sample of military recruits based on the possibility that recruits may not have prior experience with load carriage. Fourth, we conducted our experiments in a controlled laboratory environment using a treadmill. While this laboratory setup is not fully representative of military training conditions during BCT, we implemented it to minimize confounding factors and systematically delineate the effect of sex on the running biomechanics. Fifth, as the primary movement of knee and ankle joints occurs in the sagittal plane, we modeled the knee and ankle joints as revolute joints, which capture their movement in the sagittal plane. Indeed, using musculoskeletal models, Marra et al. (2015) evaluated the performance of revolute and higher-fidelity joints in their ability to represent knee joint biomechanics and found their performance to be comparable. Therefore, we believe that including the 3-D motion of the knee and ankle joints would not have changed our conclusions. Sixth, in addition to optimizing the length of body segments using marker data, we used the AnyBody built-in adjustment based on the work of Frankenfield et al. (2001), which depends on BMI, to scale the body segments in our musculoskeletal models. While using this built-in adjustment is a limitation in our study, we believe that it will not affect our findings because the formulations established by Frankenfield et al. (2001) adequately describe the relation between BMI and body fat percentage. Finally, as we pointed out in our prior studies (Unnikrishnan et al., 2021; Tong et al., 2023), our risk-prediction model assumed uniform bone adaption and repair, which were fixed for all participants. Future model enhancements that incorporate individualized bone adaption and repair processes could potentially enhance the accuracy of subject-specific SF risk predictions.

In summary, we quantified the running biomechanics of 41 young, healthy participants (20 women and 21 men) under three different load conditions and evaluated sex-specific differences in joint kinetics and kinematics, as well as the associated risk of tibial SF during a 10-week U.S. Army BCT. When running with no load, women and men displayed significantly different joint kinematics at the trunk, hip, and knee. While men tend to flex their trunk more during running, women tend to flex their hip more instead. Not surprisingly, we found that women had a significantly greater increase in lower-extremity JRFs and tibial SF risk than men as load increased. These sex-specific differences in running biomechanics explain, in part, the higher incidence rate of lower-extremity injuries in women during BCT.

## Data Availability

The datasets presented in this article are not readily available because a written request to the corresponding author along with a summary of the planned research are required to obtain the datasets and related analyses. Requests to access the datasets should be directed to jaques.reifman.civ@health.mil.
